# Social psychiatry lives!

**DOI:** 10.1192/bjb.2022.77

**Published:** 2023-04

**Authors:** Peter Huxley, Rob Poole

**Affiliations:** Bangor University, Bangor, UK

**Keywords:** Social psychiatry, distress, mental health services, psychosis, social outcomes

## Abstract

In this journal Ikkos examines the work of the American sociologist Owen Whooley, who argues that US psychiatry has gone through five paradigm shifts without defining the object of its own expertise. We look at the substance of Whooley's methods and assumptions and offer our observations on Ikkos's argument and conclusions.

We strongly support George Ikkos in his efforts to engage constructively with the social sciences and with their critiques of psychiatry.^1^ His article examines the work of Owen Whooley,^2^ an American sociologist who argues that US psychiatry has gone through five paradigm shifts without ever defining the object of its own expertise. Ikkos concludes that psychiatry's object is to intervene in ‘affect’. He cites Pressman's^3^ opinion that the implicit object of psychiatry is ‘the management of despair’. Both Ikkos and Pressman come close to Goldberg & Huxley's view^4^ that distress is the main criterion for doctors and patients alike when deciding whether psychiatric help is required, and that passage through service filters is mainly determined by the degree of that distress.

In this commentary, we look at the substance of Whooley's methods and assumptions and offer our observations on Ikkos's argument and conclusions.

## Different paradigms or different models?

Whooley comes to the view that psychiatry has experienced five paradigm shifts over 200 years through his examination of the content of the *American Journal of Psychiatry* (known as the *American Journal of Insanity* until 1921). Whooley describes the authors of the journal as the ‘literary elite’ of psychiatry, the content being produced by senior clinicians, academic researchers, research funders and journal editors rather than by front-line staff with high levels of patient contact (who, of course, are not only psychiatrists). We acknowledge the pattern of radical change in the perspectives of the American psychiatric elite, but these were not necessarily ubiquitous or sequential among mental health practitioners, including practising psychiatrists.

Throughout medicine, the relationship between medical science and clinical practice is complex. The changes that Whooley describes in US psychiatry are sudden changes in the dominant treatment model rather than scientific paradigm shifts as described by Kuhn.^5^ Furthermore, in clinical medicine (as opposed to medical science), change is slow and occurs in response to many contextual factors other than the intellectual frameworks employed by elites. In the UK, Meyer's psychobiology, although rarely referred to by name, has had an enduring effect through the pervasive influence of Sir Aubrey Lewis after the Second World War. Lewis supported Maxwell Jones in developing therapeutic communities at Belmont Hospital, Sutton, and Jones went on to pioneer community psychiatry^6^ at Dingleton Hospital in Melrose. The legacy of all three developments coexist in modern British psychiatry. They do not deploy different paradigms; they are different applications of a single paradigm.

## Determining the object of expertise: clinicians versus funders

In the UK, different models have not necessarily been in competition for dominance, as is illustrated by the eclectic nature of successive Royal College of Psychiatrists curricula for psychiatric trainees. Whooley follows the common *a priori* assumption among critics of psychiatry that the discipline stands outside of, and distinct from, the main body of medicine. US medicine, he claims, has had just one paradigm shift that led non-psychiatric doctors to a constant and steadfast object of their expertise, which is an unwarranted assertion in our view. In fact, scientific uncertainty and major gaps in understanding (described as ‘ignorance’ by Whooley) are pervasive throughout medicine, as is controversy over the purpose of treatment. All of medicine contains a tension between the objective of easing *suffering* and curing *disease*, given that success in treating infectious disease with antimicrobials has not been matched by new treatments for other types of disorder. There are few medical treatments where the balance of desired and unwanted effects can be appraised through the application of a mathematical equation. For example, in pain medicine, opioids are powerful analgesics that ease suffering, but nonetheless, chronic pain cannot be eliminated through their use; what then is the legitimate purpose of opioid medication and what outcomes are desirable? How should we assess the balance of benefits and adverse effects (individual and social) of opioid treatment? These questions are very similar to those confronting 21st-century psychiatry, which similarly has many partially effective treatments with the potential to cause marked adverse effects.

All branches of medicine are subject to the same external, environmental, structural and financial constraints and influences. These are the major determinants of radical change in models of care. We suggest that any uncertainty within psychiatry about the object of its expertise plays a minor role at most. For instance, the changes in the case management system in the USA and the desire of payors to control the distribution of funds contributed to the development of fully operationalised diagnostic systems that could be understood and administered by non-clinicians. Operationalised diagnostic criteria had existed for some considerable time before the concept fell on the fertile ground of insurers’ financial interests. One impact of the application of diagnostic criteria in clinical services has been to limit payments for certain diagnostic categories. The USA has a mixed economy of provision, dominated by private insurance, and the UK has moved steadily in the same direction under successive administrations. Large parts of National Health Service (NHS) mental healthcare is now contracted out to private services, with the most seriously unwell predominantly cared for in private locked facilities. Both Whooley and Ikkos rightly highlight the neglect of people with chronic psychosis.

Far from standing aloof from neoliberal distribution of healthcare resource, the rest of medicine is affected in similar ways to psychiatry. The impact of commodification of pain relief in the USA has been well documented under the label of ‘the opioid crisis’.^7^ In the dominant assess–treat–discharge model of care that is favoured by commercial funders, and increasingly by UK state funders, the best outcomes in the management of pain are hard to achieve because the service model neglects engagement with patients and families. It is difficult within a therapeutic relationship to take time to understand the complex mix of internal and environmental causal factors leading to distressing pain in order to facilitate change and functional improvement. The failure to deliver satisfactory outcomes is less a consequence of the scientific or conceptual limitations of pain medicine, and more a consequence of the economic model of delivery of healthcare. Exactly the same problems of the health system are evident in US and British psychiatry.

## Failures of social psychiatry and psychiatry's disengagement from it

It is the case that psychiatry, perhaps more than other specialties, has been subject to waves of overoptimism and overinvestment in solutions that have failed to fully deliver the promised benefits. The disappointing rewards of the US ‘Decade of the Brain’ have been matched by similar disappointments in social approaches when implemented formulaically at a national level. In the UK, the policy of community psychiatry was not underpinned by a coherent model, and it has degenerated into patchwork of separate individual services, each guarded by clinical criteria that exclude significant numbers of people with complex mental health needs. Even the acclaimed Italian example of the 1978 Basaglia Law (Law 180) did not deliver quite the national revolutionary change that was promised. It was intended to abolish mental hospitals and oppressive mental healthcare.^8^ Although Trieste developed exemplary community services under Basaglia's influence, this is not true of all parts of Italy.^9^ Despite its status as a World Health Organization Collaborating Centre, the Trieste service is under severe attack by a right wing regional administration.

In our opinion, Ikkos's identification of affect as the object of psychiatry is too limited, as this locates distress exclusively in the individual. Indeed, all of medicine has the relief of distress as its object. Distress is often located outside of the individual who is diagnosed with a medical disorder; for example, in paediatrics, distress is often experienced by parents rather than patients. Psychiatry exists as a distinct specialty because it specialises in relieving distress arising from disorders that are characterised by behaviour that is normatively regarded as irrational. Most of the special controversy over the discipline arises from the consequent ambiguity regarding the boundary between the relief of suffering and social control. Psychiatry needs social sciences to help it to understand and guard that boundary.

There is no doubt, as Ikkos suggests, that we need a more constructive alliance between social and medical sciences.^10^ On leaving Manchester for the Institute of Psychiatry in London, Professor Sir David Goldberg was asked if he had any regrets; he replied that psychiatry should have fought harder to retain psychiatric social work rather than allow it to be subsumed under a generic approach. In other countries, psychiatric social work continues to exist but it has disappeared as a specialty in England and Wales, where the distinctive and essential contribution of social work was effectively brushed aside when ‘approved social workers’ were replaced by ‘approved mental health professionals’ in the 2007 amendments to the Mental Health Act 1983.

In our opinion, the deficit in psychiatry lies neither in a lack of understanding (which no amount of new knowledge will ever eliminate) nor in inadequate engagement with neuroscience (which has sometimes acted as a vigorous tail wagging the psychiatric dog). Instead, psychiatry's main problem is its disengagement from social science, both theoretical and empirical, and from social practice. In contemporary clinical practice, where social intervention does occur, it is often reduced to social prescribing, an oxymoronic neologism because prescribing is individual but social is collective; the semantic contradiction flags strict limitations as to what can be achieved.

## Neoliberalism versus cost-efficiency

Ikkos is entirely correct that our socially decontextualised NHS mental health services are in a parlous state. Neoliberalism holds that economic growth is the greatest public good, but the philosophy fails to recognise capitalism's need for good public services. This need became apparent during the industrial revolution when social inequality started to undermine the efficiency of the economy. Application of neoliberal managerialism to health services has catastrophically optimised Tudor Hart's inverse care law, whereby the greatest health resource is directed at those with the least need, namely the wealthy and healthy. People with severe mental illness are neglected or, in the case of the USA, abandoned. In a review of 21st-century research, we found that clinical outcomes for psychosis have improved, but only for those experiencing a first episode. People experiencing recurrent psychosis have seen no improvement in outcomes. We suggest that early intervention is attractive to funders as a discrete, apparently curative, intervention. People with recurrent psychosis, who require longer-term, multiphasic interventions, are perceived as a drain on resources. For the neoliberal state, the issue is how to manage them cost-efficiently. Measurement of social outcomes is neglected both in the scientific literature and by services. Long-term neglect or incarceration of people with psychosis can only appear appropriate if social outcomes are not measured or analysed.

## Forging a new identity

Ikkos calls for psychiatry to forge a new identity. He implies that this should be more clearly social in nature, and we strongly support that proposition. Psychiatry and social science both work to understand and address the consequences of social adversity and injustice, even if psychiatry is sometimes reluctant to acknowledge this. Psychiatry has responsibilities at both population and individual levels. It is a fruitless enterprise to address the medical without attention to the social, but it is equally fruitless to suggest social solutions without attention to the actual illness experience and the relief of distress. As three eminent social psychiatrists suggested 10 years ago: ‘psychiatry may have been at its most attractive as a profession and most productive at times when the social perspective was fully embraced as central to it’.^11^

## Data Availability

Data availability is not applicable to this article as no new data were created or analysed in this study.

## About the authors

**Peter Huxley** is Professor of Mental Health Research, Centre for Mental Health and Society, Bangor University, Bangor, UK. **Rob Poole** is Professor of Social Psychiatry, Centre for Mental Health and Society, Bangor University, Bangor, UK.
